# Considerations for three-dimensional image reconstruction from experimental data in coherent diffractive imaging

**DOI:** 10.1107/S2052252518010047

**Published:** 2018-09-01

**Authors:** Ida V. Lundholm, Jonas A. Sellberg, Tomas Ekeberg, Max F. Hantke, Kenta Okamoto, Gijs van der Schot, Jakob Andreasson, Anton Barty, Johan Bielecki, Petr Bruza, Max Bucher, Sebastian Carron, Benedikt J. Daurer, Ken Ferguson, Dirk Hasse, Jacek Krzywinski, Daniel S. D. Larsson, Andrew Morgan, Kerstin Mühlig, Maria Müller, Carl Nettelblad, Alberto Pietrini, Hemanth K. N. Reddy, Daniela Rupp, Mario Sauppe, Marvin Seibert, Martin Svenda, Michelle Swiggers, Nicusor Timneanu, Anatoli Ulmer, Daniel Westphal, Garth Williams, Alessandro Zani, Gyula Faigel, Henry N. Chapman, Thomas Möller, Christoph Bostedt, Janos Hajdu, Tais Gorkhover, Filipe R. N. C. Maia

**Affiliations:** a Laboratory of Molecular Biophysics, Department of Cell and Molecular Biology, Uppsala University, Husargatan 3 (Box 596), SE-751 24 Uppsala, Sweden; b Biomedical and X-ray Physics, Department of Applied Physics, AlbaNova University Center, KTH Royal Institute of Technology, SE-106 91 Stockholm, Sweden; c University of Oxford, UK; d ELI Beamlines, Institute of Physics, Czech Academy of Science, Na Slovance 2, CZ-182 21 Prague, Czech Republic; e Condensed Matter Physics, Department of Physics, Chalmers University of Technology, Gothenburg, Sweden; f Center for Free-Electron Laser Science, DESY, Notkestrasse 85, 22607 Hamburg, Germany; g European XFEL GmbH, Holzkoppel 4, 22869 Schenefeld, Germany; h Linac Coherent Light Source, SLAC National Accelerator Laboratory, Stanford, California 94309, USA; i Institut für Optik und Atomare Physik, Technische Universität Berlin, Hardenbergstr. 36, 10623 Berlin, Germany; j Chemical Sciences and Engineering Division, Argonne National Laboratory, 9700 South Cass Avenue, Lemont, IL 60439, USA; k PULSE Institute and SLAC National Accelerator Laboratory, 2575 Sand Hill Road, Menlo Park, CA 94025, USA; l Division of Scientific Computing, Department of Information Technology, Science for Life Laboratory, Uppsala University, Lagerhyddsvägen 2 (Box 337), SE-751 05 Uppsala, Sweden; m Department of Physics and Astronomy, Uppsala University, Box 516, SE-751 20 Uppsala, Sweden; n NSLS-II, Brookhaven National Laboratory, PO Box 5000, Upton, NY 11973, USA; o Research Institute for Solid State Physics and Optics, 1525 Budapest, Hungary; p Department of Physics, Northwestern University, 2145 Sheridan Road, Evanston, IL 60208, USA; q NERSC, Lawrence Berkeley National Laboratory, 1 Cyclotron Rd, Berkeley, CA 94720, USA

**Keywords:** XFELs, Melbournevirus, coherent diffractive imaging, LCLS, image reconstruction

## Abstract

Diffraction before destruction using X-ray free-electron lasers (XFELs) has the potential to determine radiation-damage-free structures without the need for crystallization. This article presents the three-dimensional reconstruction of the Melbournevirus from single-particle X-ray diffraction patterns collected at the LINAC Coherent Light Source (LCLS) as well as reconstructions from simulated data exploring the consequences of different kinds of experimental sources of noise. The reconstruction from experimental data suffers from a strong artifact in the center of the particle. This could be reproduced with simulated data by adding experimental background to the diffraction patterns. In those simulations, the relative density of the artifact increases linearly with background strength. This suggests that the artifact originates from the Fourier transform of the relatively flat background, concentrating all power in a central feature of limited extent. We support these findings by significantly reducing the artifact through background removal before the phase-retrieval step. Large amounts of blurring in the diffraction patterns were also found to introduce diffuse artifacts, which could easily be mistaken as biologically relevant features. Other sources of noise such as sample heterogeneity and variation of pulse energy did not significantly degrade the quality of the reconstructions. Larger data volumes, made possible by the recent inauguration of high repetition-rate XFELs, allow for increased signal-to-background ratio and provide a way to minimize these artifacts. The anticipated development of three-dimensional Fourier-volume-assembly algorithms which are background aware is an alternative and complementary solution, which maximizes the use of data.

## Abstract 2

A three-dimensional reconstruction of the Melbournevirus affected by a strong artifact in the center of the particle is presented. Using simulations, the artifact was found to be probably caused by background scattering, while particle size and pulse-energy variation did not affect the quality of the reconstruction. Possible ways to minimize such problems in the future are suggested.

## 1. Introduction   

X-ray crystallography has for decades been the major technique to solve the structure of proteins, but one of the bottlenecks is the production of well-diffracting crystals. The method of flash X-ray imaging (FXI) aims to record diffraction directly from single macromolecules (Neutze *et al.*, 2000 ▸) and has the potential to allow structure determination of biological particles without the time-consuming crystallization step. By studying single particles it is possible in principle to capture conformational substates that would otherwise not be detectable from the averaged ensemble of a crystal. Once the technique matures, there is a tremendous potential in FXI to study conformational heterogeneity as well as time-resolved structural changes of biological samples (Spence, 2017 ▸). Instead of getting the diffraction signal enhanced by repeating units of the protein in a crystal into strong Bragg peaks, FXI leans on a sufficiently bright X-ray pulse. Unfortunately, the high-intensity X-ray radiation needed to measure a single particle’s diffraction pattern completely obliterates the sample because of radiation damage. Theoretical work has shown that femtosecond pulses of intense X-ray radiation produced by free-electron lasers can outrun the key radiation-damage processes (Neutze *et al.*, 2000 ▸; Jurek *et al.*, 2004 ▸; Bergh *et al.*, 2008 ▸) and produce a diffraction pattern of a practically damage-free particle. This ‘diffraction before destruction’ method was successfully employed experimentally for the first time in 2006 ▸ (Chapman, Barty, Bogan *et al.*, 2006 ▸). Since then the technique has been used to determine low-resolution two-dimensional images from single diffraction patterns of several biological samples including cells (Mancuso *et al.*, 2010 ▸; Seibert *et al.*, 2010 ▸; Schot *et al.*, 2015 ▸), cell organelles (Hantke *et al.*, 2014 ▸) and virus particles in two dimensions (Seibert *et al.*, 2011 ▸; Kassemeyer *et al.*, 2012 ▸; Daurer *et al.*, 2017 ▸) and three dimensions (Ekeberg *et al.*, 2015 ▸; Kurta *et al.*, 2017 ▸).

In FXI, only the modulus squared of the Fourier transform of the particle can be measured and the phase of the diffracted wavefunction has to be determined in order to reconstruct the object. The diffraction pattern of a single particle is continuous, which makes it possible to determine the phase computationally through an iterative process (Fienup, 1978 ▸). Two constraints are enforced in iterative phase retrieval. The first constrains the Fourier amplitudes to be consistent with the experimental intensities and the second constrains the object to reside within a limited real-space volume, also known as the support (Marchesini *et al.*, 2003 ▸).

For three-dimensional imaging there is the additional problem of the unknown orientations of the particles, which have to be retrieved in order to build up a three-dimensional diffraction volume from which a three-dimensional image can be reconstructed (Loh *et al.*, 2010 ▸). Since the particles arrive in the X-ray focus with a random orientation, the rotations have to be determined computationally from the noisy diffraction patterns. Imaging in three dimensions requires sample homogeneity, either within the full data set or a homogeneous subset of the data (Maia *et al.*, 2009 ▸), and thus requires careful image selection in order to obtain a good reconstruction. The rotation problem as well as the requirement for homogeneity of the image set makes three-dimensional imaging with FXI more challenging than the single-shot two-dimensional imaging case, which can be performed on non-reproducible objects.

In the experiment, single particles are typically introduced into the X-ray beam as a stream of free particles in a vacuum, thereby circumventing the need for a substrate that would yield unnecessary background scattering. Even so, background scattering from optical components of the beamline as well as from gas used during sample delivery is still present in the data. Assuming the sample is homogeneous, the strength of the signal can be increased, compared with the background, by averaging diffraction patterns with identical orientations (Huldt *et al.*, 2003 ▸).

In this article, we present a three-dimensional reconstruction of a 230 nm icosahedral virus, the Melbournevirus (MelV) at 28 nm resolution from experimental FXI data. The reconstruction contains a central region with twice the density of the surrounding particle. This difference is larger than what would be expected from any biological sample, pointing to it being a reconstruction artifact. To understand what may cause this artifact, we study the effects that background, sample heterogeneity and diffraction-space blurring have on the quality of FXI three-dimensional reconstructions using simulated data.

## 2. Methods   

### 2.1. Data collection   

The experiment was performed with the LAMP instrument (Ferguson *et al.*, 2015 ▸) at the AMO endstation (Bozek, 2009 ▸) of the LINAC Coherent Light Source (LCLS) free-electron laser (Emma *et al.*, 2010 ▸). The sample was aerosolized with a gas-dynamic virtual nozzle (GDVN) and delivered into the X-ray focus as a stream of isolated particles using a purpose-built aerosol particle injector (Seibert *et al.*, 2011 ▸; Hantke *et al.*, 2014 ▸). Far-field diffraction of the aerosolized particles was detected by a pnCCD detector (Strüder *et al.*, 2010 ▸) positioned 0.732 m downstream of the interaction region and monitored using *Hummingbird* (Daurer *et al.*, 2016 ▸). The detector consisted of two detector halves separated by 1.19 mm with a total size of 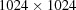 pixels and a pixel size of 75 × 75 µm. Data were recorded during an X-ray Fourier holography experiment (Gorkhover *et al.*, 2018 ▸), where Xe clusters with an average diameter of 30–120 nm were used as holographic reference and were produced at 10–30 Hz *via* a supersonic expansion from a separate cluster injector. The free-electron laser was operated at 1.2 keV photon energy (corresponding to an X-ray wavelength of about 1.0 nm) producing ∼100 fs long pulses of ∼2 mJ at 120 Hz repetition rate. The X-ray beam was focused by a pair of Kirkpatrick–Baez mirrors down to nominally ∼2 × 2 µm in order to achieve intensities at the interaction region necessary for FXI. The actual intensity on the sample was estimated to 0.01 mJ µm^−2^ by performing a low-resolution spherical fit according to §2.4 and described in detail in Daurer *et al.* (2017 ▸).

### 2.2. Data preprocessing   

Preprocessing of the raw data was performed with the *Cheetah* software package (Barty *et al.*, 2014 ▸). The relative positions of the detector halves with respect to the X-ray beam axis were recovered from diffraction data of Xe clusters with intense and sharp Newton rings covering both halves, which yielded a horizontal separation of 1.19 mm and a vertical displacement of 0.24 mm. Electronic noise was accounted for by (i) subtracting a dark-frame average of ∼4000 frames recorded prior to the X-ray exposure, and (ii) performing a common-mode correction where the zero-photon peak was determined individually for each line in the detector halves and subtracted on a shot-by-shot basis. Polarization correction and solid-angle correction were performed as described in Sellberg *et al.* (2014 ▸). Additionally, a photon background (created from the median intensity of the last 100 non-hit frames using *Cheetah*’s running background algorithm), consisting of stray X-rays not properly focused, was subtracted. Finally, defective pixels with non-linear detector responses were masked out, as determined by a pixel-by-pixel gain map with a nominal gain of 95 analog-to-digital units (ADU) per photon. In particular, saturated pixels (above 10 000 ADU), hot pixels (90% of the last 100 frames above 5000 ADU) and noisy pixels (whose standard deviations of the last 100 background frames are above 500 ADU) were also considered defective.

### 2.3. Selection of diffraction patterns   

**Figure 1:**
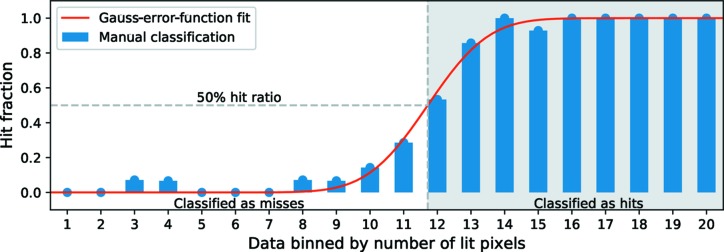
Lit-pixel threshold determination for a typical recorded run. The first 287 patterns from the run were manually classified into hits and misses. Those patterns were then sorted, by the number of lit pixels, into 20 bins. The hit fraction for each bin is shown along with a fitted Gauss-error function. The point at which the fit reaches a hit fraction of 0.5 determines the lit-pixel threshold used for automatic hit finding in the *Cheetah* software package (Barty *et al.*, 2014 ▸).

Hit selection occurred in multiple steps. In the first stage we used a threshold on the number of lit pixels to find hits. Pixels with a value above 250 ADU, corresponding roughly to 3-photon events or more, were considered lit. The minimum number of lit pixels required to consider an image a hit was set individually for every recorded run as shown in Fig. 1 ▸. The first ∼300 shots of each run were manually classified through visual inspection. A hit was here defined as any visible diffraction above the persistent-stray-light background produced by upstream beamline components. Those ∼300 shots were then sorted by the number of lit pixels into 20 bins and the hit fraction was calculated for each bin. One expects the bins with few lit pixels to have almost no hits and *vice* *versa*. To define the lit-pixel threshold we fitted the fraction of lit pixels using the Gauss-error function and selected a threshold corresponding to the number of lit pixels at which the Gauss-error function reached half its maximum (its inflection point), as illustrated in Fig. 1 ▸.

Data were recorded in 56 runs during two shifts, resulting in 965 739 hits at 42.9% hit rate. The X-ray background was greatly different between the two shifts, which was reflected in the lit-pixel threshold (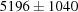 lit pixels and 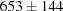 lit pixels, respectively). Thus, only hits from the second shift with lower background were further processed for subsequent image selection.

The full experimental data set contained MelV single hits, MelV multiple hits, aggregates, Xe cluster hits and Xe + MelV holographic hits. Only MelV single hits can be used for three-dimensional reconstruction, which necessitates further image selection. Three different image selections were performed on the full experimental data set and used for reconstructions. The first image selection was performed by manually selecting 670 patterns, picking those resembling diffraction from an icosahedron, from only one run containing 10 303 hits that was collected during ∼2 h. The second selection was done on the entire shift during which the background was lower. Firstly, 2691 patterns were automatically picked from 817 976 hits and secondly from this set a subset of 586 patterns were manually selected. The automatic picking was performed with the *Redflamingo* software, which selected strong patterns estimated to originate from an icosahedral particle. A third image selection was performed to further reduce the sample heterogeneity in the data set. This was done by selecting 260 patterns that had an estimated size closest to 214 nm, the most frequent size after the second image selection. This was the smallest number of patterns that resulted in a successful reconstruction.

### 2.4. Size estimate of experimental data   

The size of the icosahedral particles was estimated directly from its diffraction pattern by finding the radius of the sphere whose diffraction most closely matches the experimental pattern at low resolution, following the procedure described in Daurer *et al.* (2017 ▸). This is justified by the fact that at very low resolution an icosahedron is well approximated by a sphere. The low-resolution sphere fit yields, in addition to the size estimate of the particle, information about the center of the diffraction pattern and intensity on the sample at the interaction region on a single-shot basis.

### 2.5. Simulation of diffraction data   

Two-dimensional and three-dimensional diffraction images were simulated using the *Condor* software (Hantke *et al.*, 2016 ▸). An electron cryomicroscopy (cryo-EM) structure of MelV (Okamoto *et al.*, 2018 ▸) was used as input for the simulations. The inside of the virus particle, not resolved in the cryo-EM model, was set to a uniform density. The relative electron density between capsid, membrane and interior of the particle was estimated from a tomographic reconstruction of MelV from cryo-EM data. 1000 diffraction images were simulated with random particle orientations and then Poisson sampled prior to their reconstruction. X-ray wavelength, pulse energy and detector distance were chosen to match the experiment.

### 2.6. Three-dimensional reconstruction pipeline   

Three-dimensional reconstructions for experimental data and simulated data were performed using the same pipeline. Two-dimensional images were oriented with the EMC algorithm (Expand, Maximize and Compress) (Loh & Elser, 2009 ▸; Loh *et al.*, 2010 ▸) using the same implementation as in Ekeberg *et al.* (2015 ▸). Both simulated and experimental data were downsampled eight times in all directions from 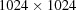 pixels to 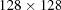 pixels prior to image orientation. Experimental data were also centered according to the diffraction-pattern center that was retrieved from the sphere fit mentioned above.

Phase retrieval in three dimensions was performed with the *Hawk* software package (Maia *et al.*, 2010 ▸). The phasing protocol started with 6000 iterations of the RAAR algorithm (Luke, 2005 ▸) enhanced with a positivity constraint (Marchesini *et al.*, 2003 ▸). The first 1000 iterations of RAAR were run with a large static spherical support with a 20-pixel radius followed by 5000 iterations with a tight static spherical support with a 12-pixel radius, corresponding to a diameter of 236 nm. The reconstruction was then refined with 1000 iterations of error reduction (Fienup, 1978 ▸). 1000 individual reconstructions were calculated and the final model is the average of these. The reproducibility of the phase retrieval was checked using the phase-retrieval transfer function (PRTF) (Chapman, Barty, Marchesini *et al.*, 2006 ▸). The resolution of the reconstruction is estimated by the point at which the PRTF falls below 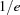 (Chapman, Barty, Bogan *et al.*, 2006 ▸; Seibert *et al.*, 2011 ▸).

### 2.7. Real-space residual   

The real-space residual (RSR) is a real-space validation tool used in X-ray protein crystallography to assess the resemblance between the electron density calculated from experimental structure factors and calculated structure factors from the model on a residue basis (Jones *et al.*, 1991 ▸). Here, the real-space residual is calculated on a voxel basis and compares the difference in electron density between the average reconstructed model (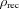) and a support object with a uniform density of one (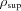). The RSR was calculated for voxels where the density of the average reconstructed model is above 10% of the maximum density according to
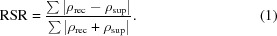



## 3. Results and discussion   

### 3.1. MelV reconstruction from experimental data   

**Figure 2:**
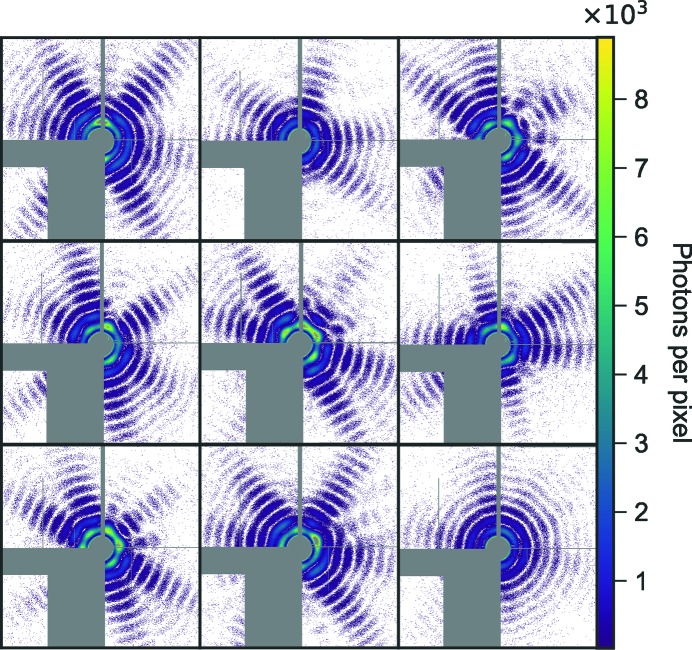
Nine high-quality diffraction patterns of MelV from the experiment. Gray areas represent masked-out regions.

A three-dimensional reconstruction from single-particle diffraction data requires sample homogeneity. Only a small fraction of the one million hits collected in this experiment are high-quality diffraction patterns of MelV. The first step was therefore to select those high-quality diffraction patterns. In this work, a high-quality diffraction pattern has a strong scattered signal and clear icosahedral features. The selected image set must also have a small size distribution, a requirement for sufficient sample homogeneity to allow for a successful reconstruction. A few examples of selected high-quality diffraction patterns are shown in Fig. 2 ▸.

**Figure 3:**
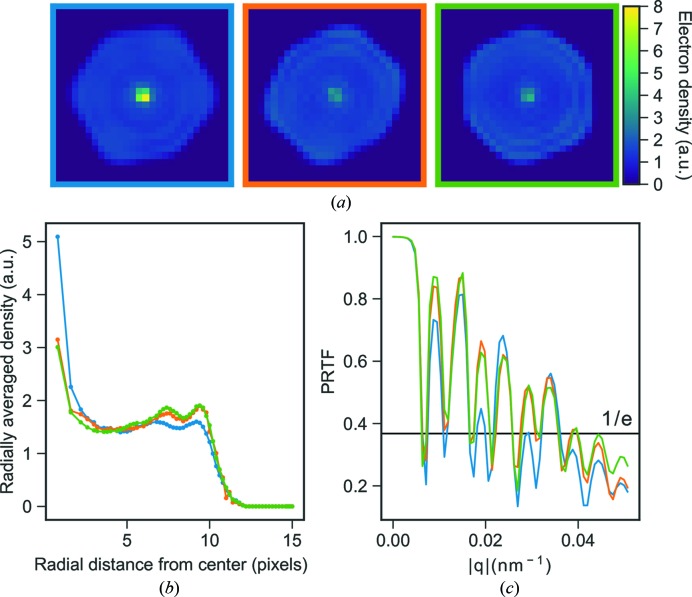
(*a*) Slices from three-dimensional reconstructions from three different image selections: a manual selection of 670 patterns from a 2 h data collection (blue), an automatic image selection of 586 patterns with a subsequent manual sub-selection (orange) and a tighter size restriction on the orange image selection consisting of 260 patterns (green). (*b*) Radial average plots of the reconstructions and (*c*) their corresponding PRTF. The colors represent the same image selection in all three sub-figures.

Three-dimensional electron densities were reconstructed from three different image selections from the experimental data. The reconstructions are shown in Fig. 3 ▸ and a striking dense feature is observed in all reconstructions covering the center 8 voxels of the reconstructions. To compare the strength of the artifact between different reconstructions a relative density (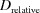) was calculated according to
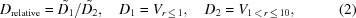
where 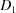 is the electron density of the voxels within the reconstructed volume (*V*) within a radius of 1 pixel from the center. 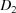 is the electron density of the voxels within the reconstructed volume within a radius of 10 pixels, excluding the voxels in 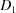. 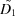 and 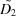 are the median of the electron density of the voxels in 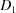 and 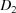, respectively.

The relative density, 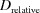, of this strong feature varies depending on the image selection but the density is always higher than any known biological feature that to our knowledge could be present in the MelV particle. The lowest relative density among these three reconstructions is 1.7, which is still high compared even to the densely packed chromatin in eukaryotic cells, at a density of 1.4 g cm^−3^ (Rickwood, 1978 ▸).

**Figure 4:**
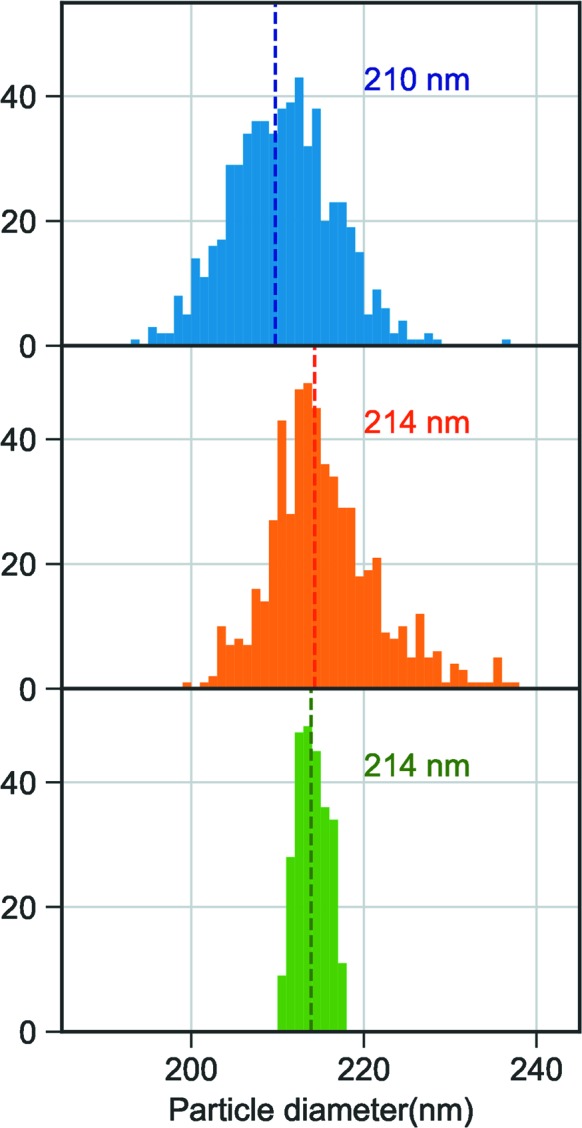
Histograms of MelV sizes retrieved through sphere fit on experimental data, comparing different image selections. Manual selection from one run (blue), automatic image selection with manual clean up (orange) and sub-selection with narrow size distribution (green). The color coding for the image selections is the same as in Fig. 3 ▸. The dashed lines in all histograms represent the median size of the distribution.

The first manual image selection of 670 patterns has a maximum size variation of 43 nm. The corresponding reconstruction has a relative artifact density, 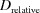, of 3.1. When instead performing the reconstruction on the second set of automatically selected patterns, 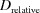 is lowered to 1.9. Further restricting the sample heterogeneity in the data set in the third image selection from 586 images with a 46 nm size variation to a set of 260 images with a maximum size variation of 6.5 nm slightly reduces 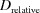 to 1.7. This shows that the central artifact can be reduced in density by employing stricter selection criteria on a larger set of images. The size selection in particular does, however, have a surprisingly small effect on the artifact. The radial densities of the reconstructions are shown in Fig. 3 ▸ *b*, where the higher density of the center is clearly visible. The observed size distributions for the image selections (Fig. 4 ▸) are wider than what can be explained by different projections of an icosahedron, jitter in the sample detector distance or the bandwidth of the X-ray free-electron (XFEL) beam.

**Figure 5:**
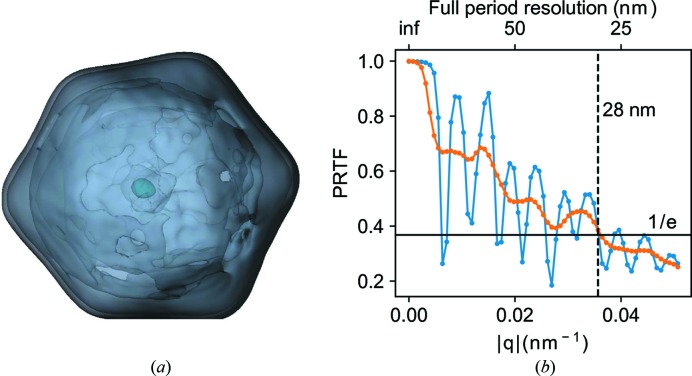
(*a*) Three-dimensional representation of the reconstruction from experimental data (a strict selection of 260 patterns with a limited size distribution). (*b*) PRTF for the reconstruction in (*a*). The blue line represents the radial average of the three-dimensional PRTF and the orange line is the critically sampled three-dimensional PRTF. The critically sampled PRTF shows a full-period resolution of 28 nm.

The only proposed explanation to such a broad size distribution is ‘caking’ where smaller chemical components in the sample solution will form a layer onto the particle after droplet evaporation (Daurer *et al.*, 2017 ▸). The reconstruction with the lowest artifact density achieved here is shown as a three-dimensional representation in Fig. 5 ▸. The reconstruction has a resolution of 28 nm according to where a critically sampled PRTF (the oversampled PRTF convoluted with a top-hat kernel as wide as a Shannon pixel) falls below 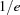 (Fig. 5 ▸ *b*) (Chapman, Barty, Bogan *et al.*, 2006 ▸).

It is clear that the strength of the artifact is correlated with the image selection, but, from the experimental data alone, it is impossible to deduce what properties in the data or problems in the reconstruction pipeline result in the observed artifact. Therefore, as a complimentary approach, we simulated diffraction patterns from an idealized MelV particle, oriented the patterns with EMC and then reconstructed the three-dimensional image, in identical fashion to experimental data, so that changes in input to EMC as well as to the phasing can be correlated with the reconstruction quality.

### 3.2. MelV reconstruction from simulated data   

Diffraction patterns from a perfectly homogeneous sample simulated with parameters matching the experiment can be easily assembled with EMC into a three-dimensional diffraction volume resembling the amplitudes of the three-dimensional Fourier transform of the object as intended. Subsequent phasing recovers the phases reliably to a full-period resolution of 20 nm. The capsid is resolved but not the inner membrane and the interior of the particle is uniform and does not contain any visible artifacts as in the case for experimental data. This result gives us confidence that the artifact is a result of the experimental data and not a result of the reconstruction pipeline itself.

### 3.3. Pulse and particle-size variation in simulations   

The patterns from the second selection of experimental data have a maximum size difference of ±20 nm around the expected size of MelV (214 nm) and are thus not a completely homogeneous data set. Furthermore, the X-ray pulses produced by the LCLS have different pulse energies because of the stochastic nature of the pulse generation and, more importantly, the X-ray focus has a spatially varying power density, which makes the intensity of the measured pattern highly dependent on the position of the particle within the focus. The collected diffraction patterns will therefore have a large variation in the total number of photons.

**Figure 6:**
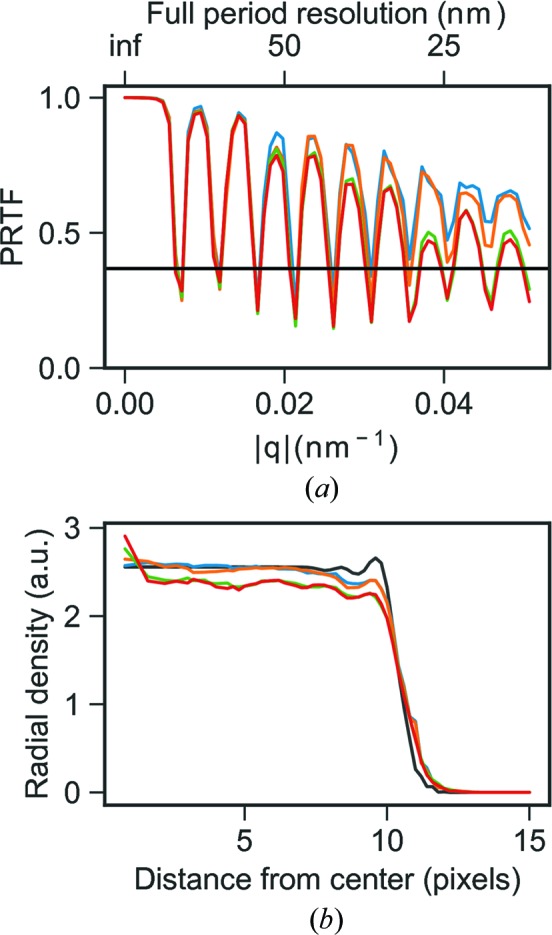
(*a*) PRTF and (*b*) radial average for three-dimensional reconstructions from homogeneous simulated data (blue), heterogeneous data (orange), homogeneous data with background (green) and heterogeneous data with background (red). The black curve represents the radial average of the input model scaled according to central density of the reconstruction from homogeneous data without added background.

The variation in number of photons in the patterns and the particle-size variation were introduced in the simulations in order to test if these factors would give rise to any artifact in the reconstructed model. The distributions of these two parameters were estimated from experimental data (second image selection) and then used as probability distributions in the simulations to produce patterns with varying combinations of both. The cryo-EM map was resampled to produce the desired-size variation. The produced patterns could be successfully oriented with EMC and the PRTF of the reconstructed model indicates a high confidence in the recovered phases (Fig. 6 ▸). The reconstruction is very similar to the reconstruction from simulated data with uniform particle size and pulse energy (Fig. 6 ▸). This clearly demonstrates that variations in particle size and pulse-energy matching the experiment do not pose a problem for three-dimensional FXI.

### 3.4. Background scattering   

**Figure 7:**
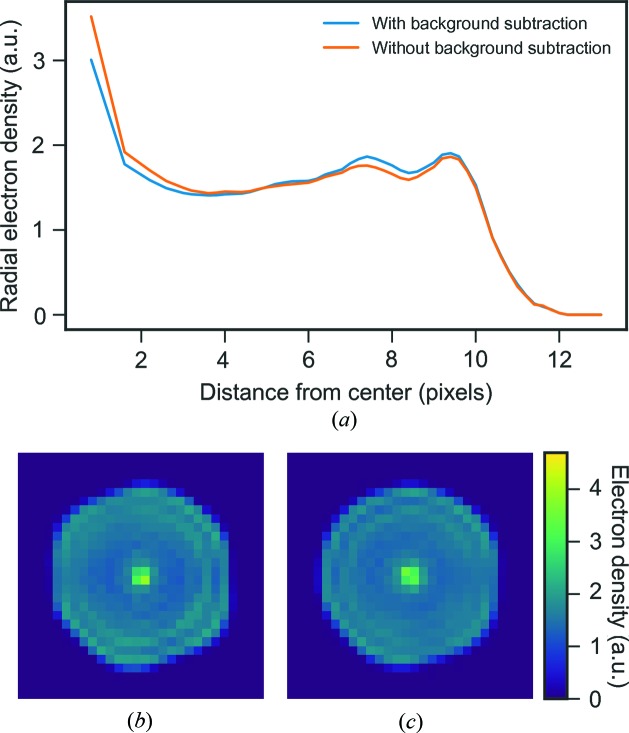
(*a*) Radial average of three-dimensional reconstructions for experimental data with (blue) and without (orange) background subtraction applied on the raw images. The images show slices through the three-dimensional reconstructions with background subtraction applied to the raw images (*b*) and without background subtraction (*c*).

One big difference between simulated data and experimental data is the presence of background scattering. Beamline components scatter and add to the background. Aerosol injection introduces focusing gas, sample buffer and sample impurities in the interaction region that all contribute to increase the background scattering. Background subtraction was performed on the diffraction images prior to image orientation and phasing for the reconstructions previously discussed. A reconstruction performed on patterns without any background subtraction has a 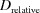 of 2.1 instead of 1.7 showing that the intensity of the background correlates with the strength of the observed artifact (Fig. 7 ▸).

Experimental background was added to the simulated data in order to explore its effects on the three-dimensional reconstructions. The experimental backgrounds were taken from a run with only buffer injected into the chamber and scaled to correct for the difference in pulse energy between the sample and buffer runs. This is the best background estimate we have, yet it probably underestimates the real background as it misses several sources of background, namely inelastic scattering from the sample and the lack of perfect coherence from the self-amplified spontaneous emission (SASE).

**Figure 8:**
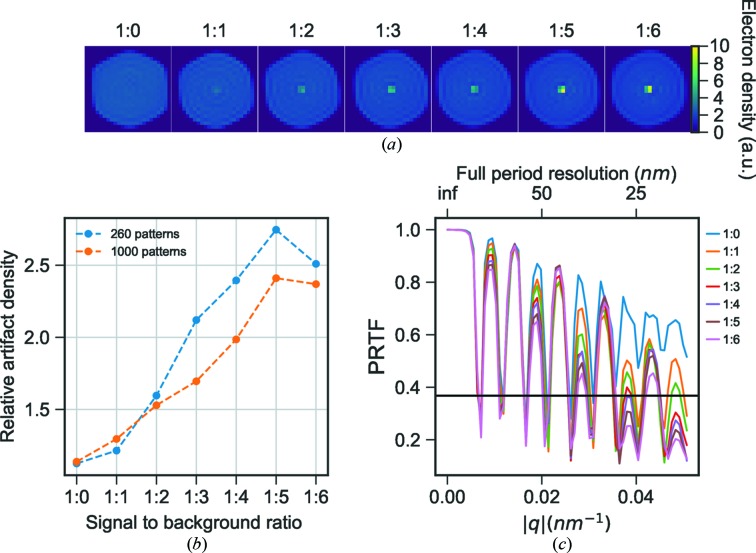
(*a*) Slices through reconstructions from simulated data with experimental background added in increasing proportions (ratio indicated above each image). (*b*) The calculated relative density of the 8 voxel central artifact compared with the median density of the particle interior for reconstructions with increasing proportion of experimental background from simulated data using 1000 (orange) and 260 (blue) images. (*c*) The PRTF for the reconstructions.

Individual backgrounds were added to 1000 simulated patterns in increasing proportion (from one up to eight times the original background signal) and then oriented with EMC. For the successful EMC runs, 1000 individual reconstructions were performed and the average reconstructed models were calculated. A strong artifact appeared in the central 8 voxels with 1.3 stronger density compared with the surrounding interior of the particle when applying the background in a 1:1 ratio. This is lower than the experimental one probably because of the above-mentioned underestimation of the real background. 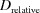 increases linearly with the background-to-sample diffraction ratio (Fig. 8 ▸ *b*). A second set of simulations was performed using only 260 images (same number of patterns as in the experimental reconstruction in Fig. 5 ▸) and the central density increases in a similar manner but with overall higher relative density (Fig. 8 ▸). When more patterns are used in the reconstruction pipeline the reconstruction is less strongly affected by background-induced artifacts since random components average out. With increasing background the PRTF shows a decay in quality, especially on every second fringe starting from the first fringe (Fig. 8 ▸ *c*), behaving similarly both when using 1000 and 260 patterns. At a 1:7 signal-to-background ratio the orientation recovery in EMC recovers features of the background and at 1:8 signal-to-background ratio EMC produces a spherically symmetric diffraction volume and completely fails to orient patterns according to the icosahedral symmetry. Disregarding the strong density in the center, all reconstructions have a similar density distribution.

The strength of the central feature for a reconstruction with only background scattering is the same as when the background scattering is combined with variation of particle size and pulse energy (Fig. 6 ▸). The central strong density is thus only affected by the addition of background and is not correlated with any heterogeneity in particle size or variation in pulse energy. This points to the artifact being the result of the Fourier transform of a relatively flat background, which will have a narrow distribution in real space.

### 3.5. Effect of blurring   

A diffracted Fourier intensity subject to background normally resembles a blurred version of the same Fourier intensities, showing decreased fringe contrast. The EMC orientation recovery might, however, also introduce a blurring, where each pattern is distributed over several orientations around the correct one. We therefore investigated the connection between intensity blurring and the reconstructed density.

**Figure 9:**
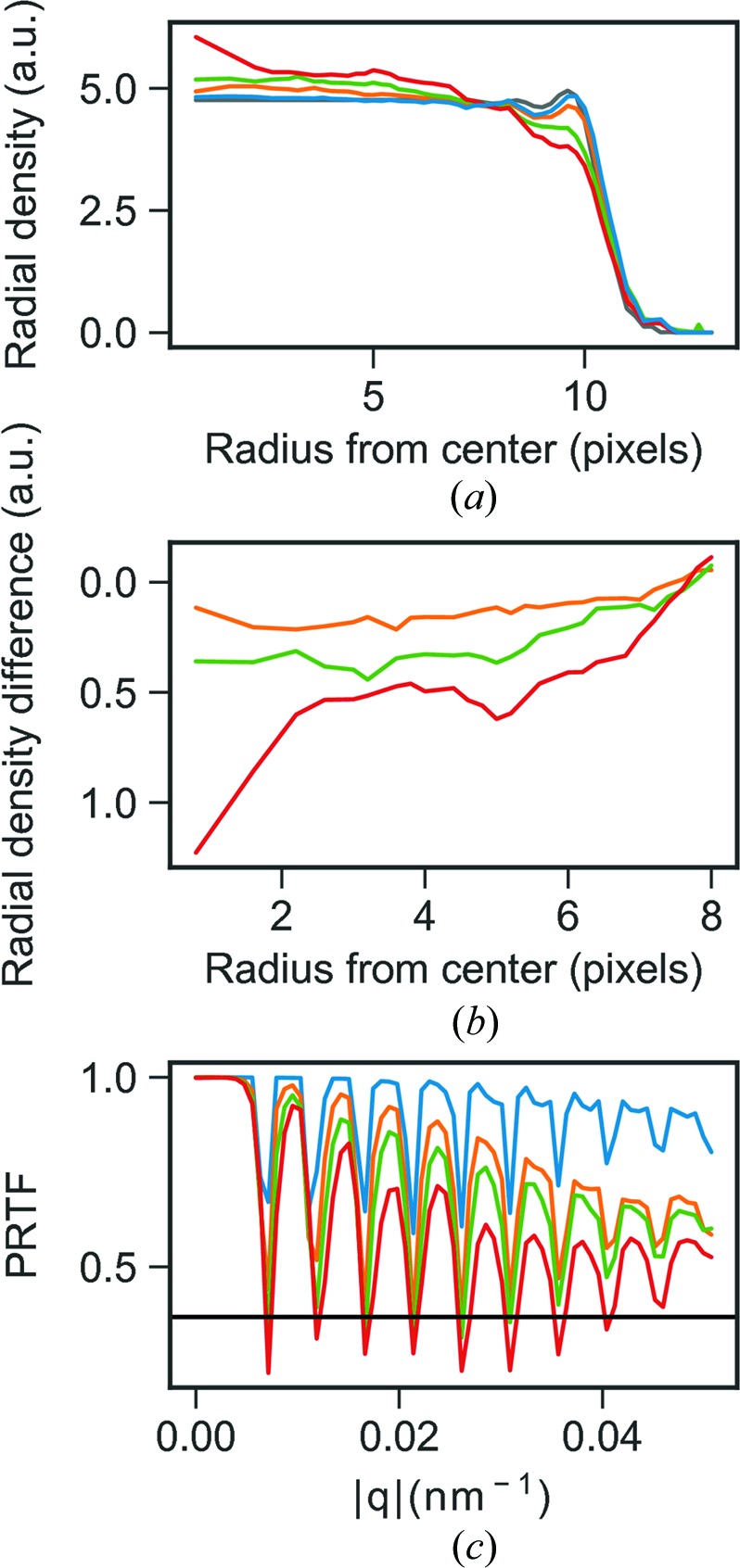
(*a*) Radial averages of reconstructions from blurred models with Gaussian blur kernels of 0 (blue), 1.0 (orange), 1.5 (green) and 2.0 (red). In gray is the radial average of the input model used in the simulations scaled to the reconstructed models. (*b*) Differences in radial averages from left plot, blur1.0 − blur0 (orange), blur1.5 − blur0 (green) and blur2.0 − blur0 (red). (*c*) PRTF for the reconstructions from blurred models with Gaussian blur kernels of 0 (blue), 1.0 (orange), 1.5 (green) and 2.0 (red).

We did this by reconstructing MelV directly from its three-dimensional diffraction volume. Blurring was introduced by convoluting the simulated three-dimensional diffraction volumes with a Gaussian kernel with a standard deviation of between 0.5 and 2 pixels in steps of 0.5. The reconstruction from a non-blurred Fourier model shows features of the capsid and a uniform interior in agreement with the input model and the PRTF shows high confidence in phase retrieval with only a slight fall-off at higher *q* (Fig. 9 ▸ *c*).

**Figure 10:**
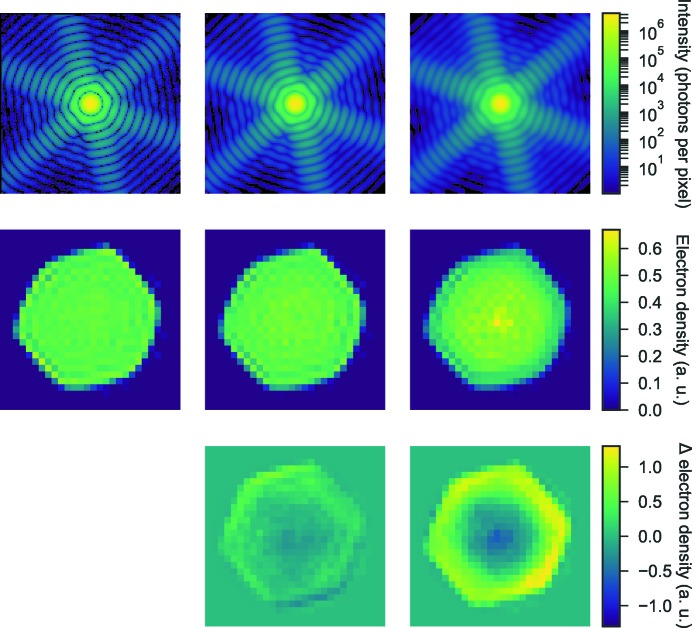
Reconstructions from blurred three-dimensional Fourier volumes. The first row of images shows one slice through the 8 times downsampled Fourier volumes in log scale with no blur (left), 1-pixel standard deviation Gaussian blur (middle) and 2-pixel standard deviation Gaussian blur (left). The second row shows slices through the corresponding three-dimensional reconstructions. The last row of images shows the difference in density between the reconstruction from the non-blurred and blurred Fourier volumes, with 1-pixel (left) and 2-pixel (right) standard deviation of the Gaussian blurring kernels.

When introducing blurring, the PRTF is lower overall for higher standard deviation of the Gaussian blur kernel and falls off with increasing *q* but only falls under 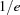 at the minima *q* positions (Fig. 9 ▸ *c*). The reconstructions from blurred diffraction volumes show increasing deviation in density from the non-blurred case. At 1-pixel blur, there is only a slight change in density which is hardly visible in a two-dimensional slice, while the reconstruction with 2-pixel blur clearly deviaties from the input model and has higher density in the center of the reconstructed object. The difference in density between the reconstruction from a non-blurred diffraction model and the blurred models is negative in the center and positive towards the edge with stronger effects the larger the blurring (Fig. 10 ▸). The average radial-density profiles of the reconstructions show that blurring in Fourier space causes the density to be redistributed from the edges to the center (Figs. 9 ▸ *a*, 9*b* and 10 ▸). The slope of the density gradient increases with larger blurring kernels. In the center of the reconstruction a 2-pixel Gaussian kernel blur introduces a 25% change in density, a 1.5-pixel blur an 8% change and a 1-pixel blur only around 2% compared with the center of a reconstruction from the non-blurred Fourier volume. It is clear from these results that blurring of Fourier space can introduce diffuse artifacts in reconstructions, an important effect to be evaluated before interpreting diffuse density changes as real features of biological significance (Fig. 10 ▸).

**Figure 11:**
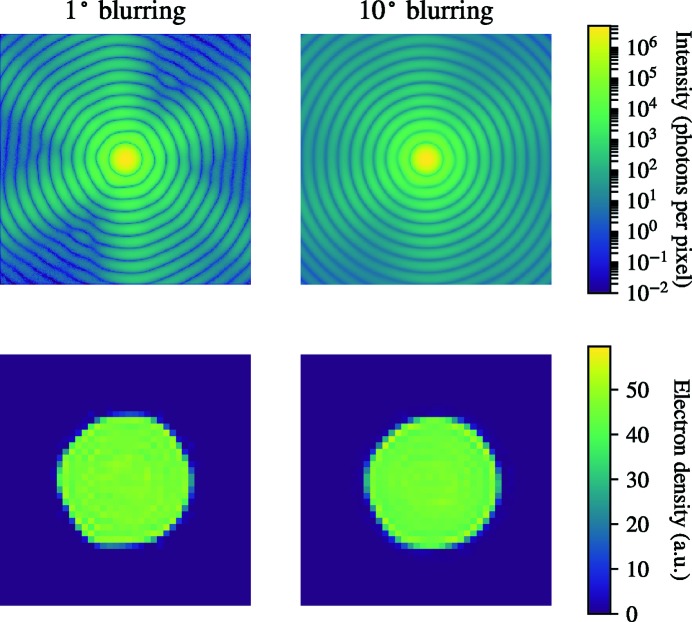
The intensity space was rotationally blurred with a standard deviation of 1 and 10°. Slices through the resulting Fourier intensities (top) are shown together with the corresponding reconstructed electron densities (bottom). This type of blurring did not produce the same type of artifacts as we saw when the blur also included the radial direction.

Interestingly, we see a similar type of effect from this artificial blurring as we do in the reconstruction of the experimental data. To distinguish whether this blurring originates from the data or from the EMC algorithm we applied a second type of blur. This time we only applied rotational blur, which simulates the kind of blurring that might be induced by the EMC algorithm. In Fig. 11 ▸ we see that the blurred Fourier intensity still maintains a high-fringe visibility even at a high degree of blurring. The reconstructions show no artifact even for a rotational blur with a standard deviation as high as 10°. The reconstruction does however, as expected, look significantly more spherical. This indicates that the dense artifact in the experimental data is not caused by the EMC algorithm but is more likely to be an effect of the experimental background.

### 3.6. Reconstruction stability   

The variation in RSR between individual three-dimensional images reconstructed from the same Fourier volume can be used to assess the reconstruction stability. The RSR calculated here is a measure on how different the reconstructions are from an object with uniform density, with lower RSR values indicating high resemblance. A similar RSR for all individual reconstructions would imply that the Fourier amplitudes given to the iterative phase retrieval converges to similar solutions and thus indicates higher reconstruction stability. The capsid and membrane present in the model particle have higher density compared with the interior that increases the total RSR value of the full particle from 0 to 0.18.

**Figure 12:**
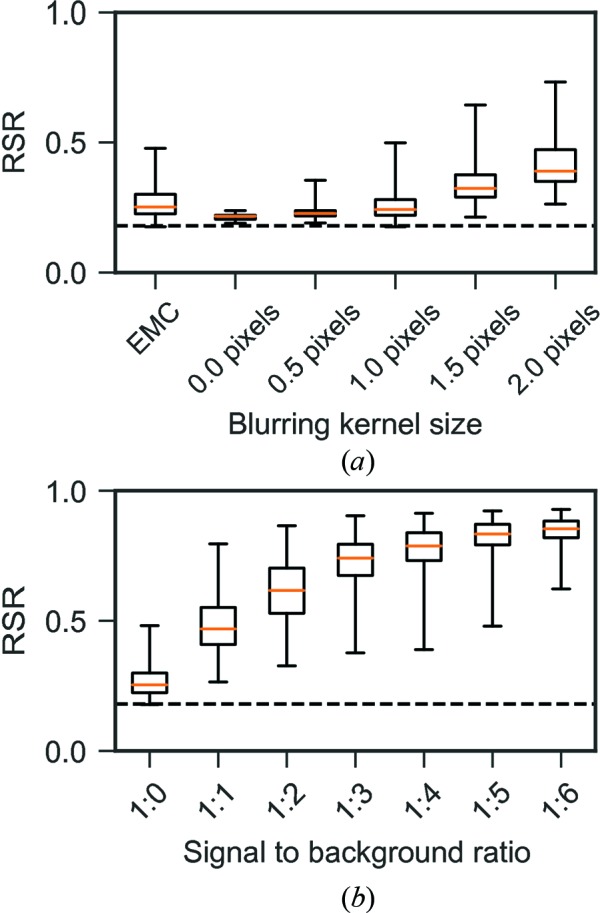
(*a*) RSR box and whisker plots for reconstructions from three-dimensional Fourier volumes blurred with a Gaussian kernel of increasing width. RSR for a model from simulated two-dimensional patterns oriented with EMC is shown as a comparison to the blurred models. (*b*) RSR box and whisker plot for reconstructions with increasing background ratio. The dashed horizontal line shows the RSR for the input model in both box plots. The mean RSR is shown with an orange line, the boxes indicate the interquartile range and the whiskers represent the lowest and highest RSR value for each group.

The RSR for reconstruction from a simulated three-dimensional diffraction volume is around 0.2 for all individual reconstructions. When blurring is added to the diffraction space, the RSR variation increases among the individual reconstructions, which indicates a less stable reconstruction. The average RSR also increases with increasing blurring because the individual reconstructions are far from being uniformly dense (Fig. 12 ▸). The RSR analysis shows that the proportion of a reconstruction containing an artifact and the strength of that artifact increase together with the standard deviation of the Gaussian blurring kernel. The reconstruction from EMC-oriented simulated patterns is similar in quality to the reconstruction from a blurred Fourier volume with a 1-pixel standard deviation Gaussian kernel, both when it comes to the stability of the reconstructions (high variation of the RSR) and to the contrast of the reconstructions (discernible capsid and interior in real-space radial average) (Fig. 12 ▸). This indicates that the impact of blurring in the final reconstruction should be negligible.

The reconstructions from EMC-oriented patterns with added experimental background show higher average RSR the stronger the background. The variance of the RSR increases only up to a background ratio of 1:4 and then decreases. For higher background ratios, where EMC has difficulty converging, the Fourier volume seems to give a more stable reconstruction (Fig. 12 ▸ *b*).

### 3.7. Post-alignment background subtraction   

To further test the hypothesis that the described artifacts are caused by background scattering we attempted to artificially remove the background from the aligned Fourier intensity volume. The background subtraction was done after the EMC step, since before the EMC step the background is sparsely sampled, just like the signal, and a background removal there could cause artifacts such as negative intensities.

We assumed a spherically symmetric background, as expected from a background dominated by scattering from residual gas. MelV is an icosahedral virus and is therefore believed to be close to centrosymmetric and with well-defined boundaries at the resolution we achieve in this article. This implies that the far-field scattered wavefunction will be close to real-valued and will cross the zero line. The measured intensities should therefore show at least one zero in every Shannon pixel.

**Figure 13:**
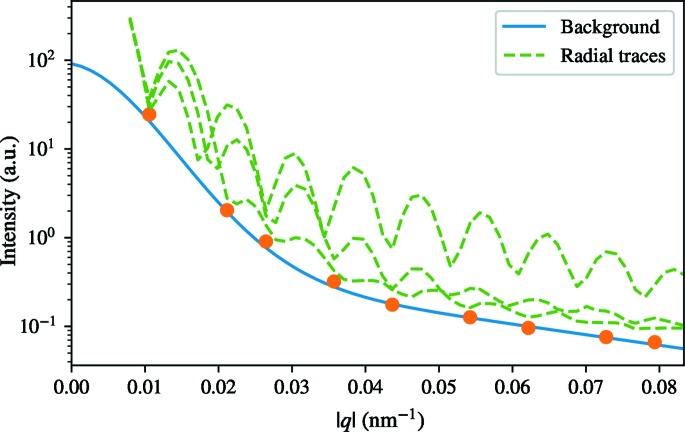
The blue line shows the radial background that was subtracted from the Fourier intensities before phase retrieval. The function is a fit to the orange points which represents the lowest points in the radial minimum function. The green lines show the values among three radial lines from the center of the Fourier intensities. As expected the background is below the signal levels at all points.

We identified these points by finding the minimum value of each radial shell of the intensities. In this function, we then found the minimum value within each Shannon pixel. These points are assumed to represent the true baseline. We then fitted a function through these points and used it as our background in the post-alignment background subtraction. We found a very good fit using the following function.
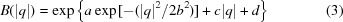
where *a*, *b*, *c* and *d* are fitting parameters. Fig. 13 ▸ shows these points together with the fitted background. It also shows three slices of the recovered patterns that show that the background is consistently lower than the intensity values.

**Figure 14:**
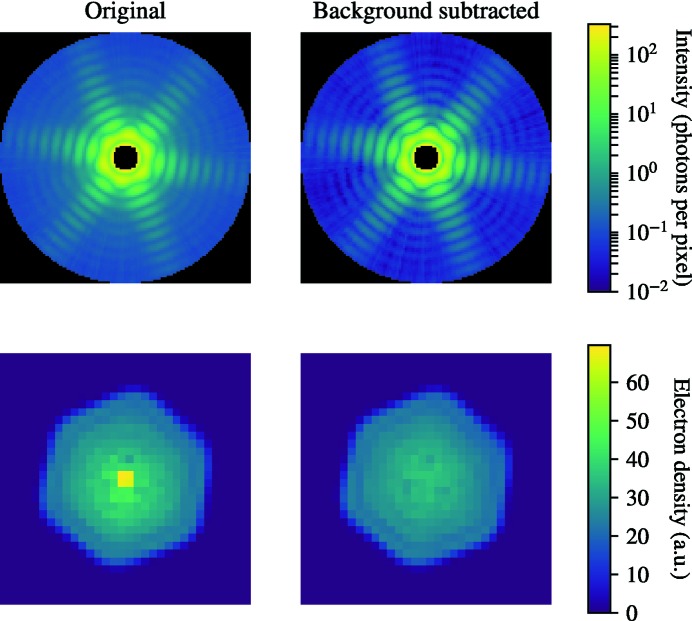
The top row shows a slice through the Fourier intensities after EMC. The left panel shows the untreated output from EMC while the right panel shows the result of the background subtraction. The background-subtracted version shows more well defined minima and clearer streaks. The bottom row shows the corresponding reconstructions. The reconstruction of the background subtracted version is very similar except that it is almost free from the strong artifact in the center that plagued the original reconstruction.

We performed phase retrieval as described in §2.6 both with and without the background subtraction and the results are shown in Fig. 14 ▸. We see that the central artifact is essentially removed by this treatment while the rest of the electron density remains largely unchanged. This supports our hypothesis that the artifact is caused by experimental background.

We do, however, stress that this method for background subtraction is not generally applicable since the assumption that the far-field wave must cross the origin is not generally true.

## 4. Conclusions   

In this article we have explored the effect of background, sample heterogeneity and blurring on the reconstructed model quality using both experimental data and simulated data. The results presented here suggest that the artifacts in the MelV models produced by FXI are mainly caused by background scattering. At the same time, care should be taken to avoid blurring which can also introduce diffuse artifacts; easily interpreted as biologically relevant density changes. On the other hand, the sample heterogeneity introduced here is not responsible for artifacts in the reconstructions and three-dimensional FXI is shown not to be as sensitive to heterogeneity as previously believed (Maia *et al.*, 2009 ▸). The quality of the model will, as is the case for all experimental techniques, be determined by the quality of the data and currently that is the main obstacle for three-dimensional FXI. Background scattering must be reduced as much as possible in the experiment to be able to achieve high-resolution, high-quality three-dimensional models. Background cannot be fully avoided and efficient strategies should be developed to take it into account during reconstruction.

A background-aware version of EMC is currently being developed. Background-aware phasing algorithms also exist (Martin *et al.*, 2012 ▸). Another route to minimize the effect of the background is to collect more diffraction patterns, enhancing the signal-to-noise ratio. This will require the high repetition rates of superconducting machines, such as the European XFEL (Altarelli *et al.*, 2007 ▸) or LCLS-II (Galayda, 2014 ▸), together with dedicated beamlines able to collect the data necessary for a high-quality model. With more data follows the need for better automated image selection and classification procedures, where machine learning with deep neural networks may provide a powerful tool.

## Supplementary Material

Fig. S1, showing lineouts through the assembled 3D intensities, and Fig. S2, showing lineouts through average background and hits. DOI: 10.1107/S2052252518010047/ec5008sup1.pdf
