# Supplementary material for: Considerations for three-dimensional image reconstruction from experimental data in coherent diffractive imaging
Source: IUCrJ. 2018 Sep 1;5(Pt 5):531–41. doi: 10.1107/S2052252518010047 (PMC6126651; doi:10.1107/S2052252518010047)
Supplement: Supplementary file 1 [file m-05-00531-sup1.pdf]

# IUCrJ

**Volume 5 (2018)**


**Supporting information for article:**

## Considerations for three-dimensional image reconstruction from experimental data in coherent diffractive imaging

**Ida V. Lundholm, Jonas A. Sellberg, Tomas Ekeberg, Max F. Hantke, Kenta Okamoto, Gijs van der Schot, Jakob Andreasson, Anton Barty, Johan Bielecki, Petr Bruza, Max Bucher, Sebastian Carron, Benedikt Daurer, Ken Ferguson, Dirk Hasse, Jacek Krzywinski, Daniel S. D. Larsson, Andrew Morgan, Kerstin Mühlig, Maria Müller, Carl Nettelblad, Alberto Pietrini, Hemanth K. N. Reddy, Daniela Rupp, Mario Sauppe, Marvin Seibert, Martin Svenda, Michelle Swiggers, Nicusor Timneanu, Anatoli Ulmer, Daniel Westphal, Garth Williams, Alessandro Zani, Gyula Faigel, Henry N. Chapman, Thomas Möller, Christoph Bostedt, Janos Hajdu, Tais Gorkhover and Filipe R. N. C. Maia**

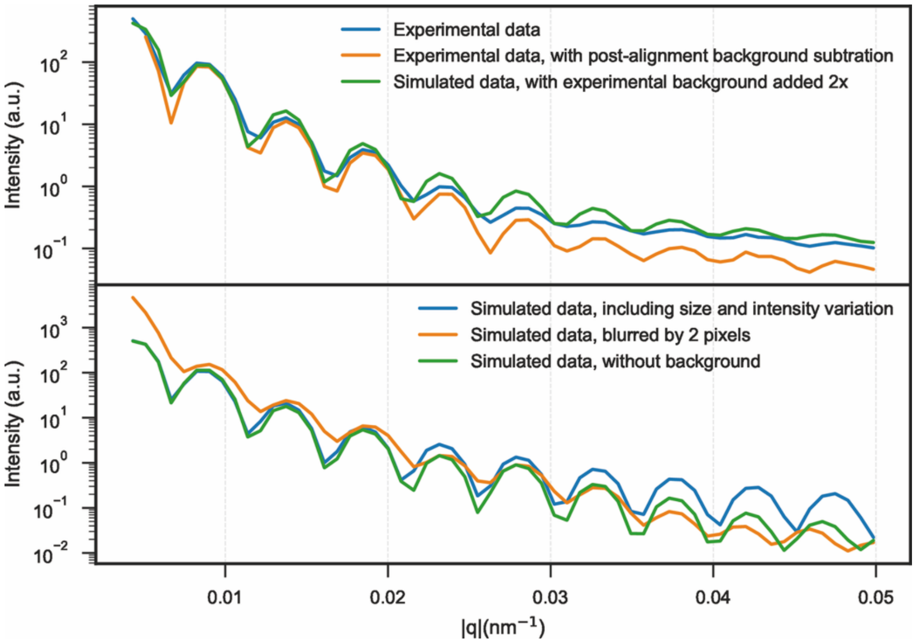


Fig. S1. Lineouts through the most representative experimental and simulated assembled 3D intensities. On the top, in blue, a lineout through the assembled model used for the reconstruction in figure 5, in orange the same data after post-assembly background subtraction as described in 3.7, and in green data simulated from the cryo-EM model with experimental background scaled by a factor of six added in. On the bottom, in blue, simulated data from the same model but taking size and intensity variation into account, in orange simulated data blurred with a Gaussian with a standard deviation of two pixels and finally in orange just the simulated data.

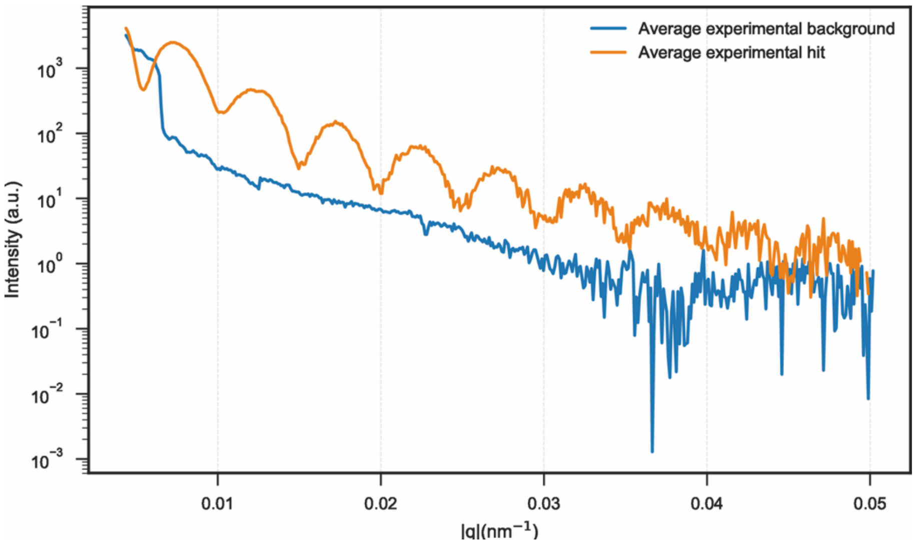


Fig. S2. Lineouts through the average of background diffraction patterns and through the average of hit diffraction patterns, after background correction. The hit diffraction patterns are those used for the reconstruction in figure 5, and the background patterns are the same as in section 3.4.
